# Stress and displacement of mini-implants and appliance in Mini-implant Assisted Rapid Palatal Expansion: analysis by finite element method

**DOI:** 10.1590/2177-6709.26.4.e21203.oar

**Published:** 2021-08-27

**Authors:** Pedro Lima Emmerich OLIVEIRA, Kayque Euclides Moreira SOARES, Rafhael Milanezi de ANDRADE, Gabriel Couto de OLIVEIRA, Matheus Melo PITHON, Mônica Tirre de Souza ARAÚJO, Eduardo Franzotti SANT’ANNA

**Affiliations:** 1 Universidade Federal do Rio de Janeiro, Departamento de Ortodontia e Odontologia Pediátrica (Rio de Janeiro/RJ, Brazil).; 2 Universidade Federal do Espírito Santo, Departamento de Engenharia Mecânica (Vitória/ES, Brazil).; 3 Pontifícia Universidade Católica, Mestrado em Ortodontia (Belo Horizonte/MG, Brazil).; 4 Universidade Estadual do Sudoeste da Bahia, Departamento de Ortodontia e Odontologia Pediátrica (Vitória da Conquista/BA, Brazil).

**Keywords:** Palatal expansion technique, Finite element method, Orthodontic anchorage procedures, Orthodontic appliances

## Abstract

**Objective:**

In this study, simulations were performed by the finite element method (FEM) to determine the tension and displacement in mini-implants and in expander appliance during rapid maxillary expansion, by varying the number and location of the mini-implants.

**Methods:**

For the computational simulation, a three-dimensional mesh was used for the maxilla, mini-implants and expander appliance. Comparisons were made on six different Mini-implant Assisted Rapid Palatal Expander (MARPE) configurations, by varying the amount and location of mini-implants. A closed suture was design and received two activations of 0.25 mm, and an open suture had a 0.5-mm aperture that received 20 activations, also of 0.25 mm.

**Results:**

For the closed suture, the maximum displacement values in the mini-implants were between 0.253 and 0.280 mm, and stress was between 1,348.9 and 2,948.2 MPa; in the expander appliance, the displacement values were between 0.256 and 0.281 mm, and stress was between 738.52 and 1,207.6 MPa. For the open suture, the maximum displacement values in the mini-implants were between 2.57 and 2.79 mm, and stress was between 5,765.3 and 10,366 MPa; in the appliance, the maximum displacements was between 2.53 and 2.89 mm, and stress was between 4,859.7 and 9,157.4 MPa.

**Conclusions:**

There were higher stress concentrations in the mini-implant than in the expander arm. In the simulations with a configuration of three mini-implants, stress overload was observed in the isolated mini-implant. Displacements of the mini-implants and arms of the appliance were similar in all simulations.

## RESUMO

**Objetivo:**

Realizar simulação pelo método de elementos finitos (MEF) para determinar a tensão e o deslocamento dos mini-implantes e do aparelho expansor durante a expansão rápida da maxila, variando a quantidade e localização dos mini-implantes.

**Métodos:**

Para realização da simulação computacional, foi utilizada uma malha tridimensional para a maxila, mini-implantes e aparelho expansor. As comparações foram feitas em seis configurações de MARPE (*Mini-implant Assisted Rapid Palatal Expander*), com quantidades e localização diferentes dos mini-implantes. Foram modeladas uma sutura fechada, que recebeu duas ativações de 0,25 mm, e outra sutura com abertura de 0,5 mm, que recebeu 20 ativações, também de 0,25 mm.

**Resultados:**

Para a sutura fechada, os valores máximos de deslocamento nos mini-implantes foram entre 0,253 e 0,280 mm, e as tensões, entre 1348,9 e 2948,2 MPa; e no aparelho expansor, os valores de deslocamento foram entre 0,256 e 0,281 mm, e as tensões, entre 738,52 e 1207,6 MPa. Para a sutura aberta, os valores máximos de deslocamento nos mini-implantes foram entre 2,57 e 2,79 mm, e as tensões, entre 5.765,3 e 10.366 MPa; e no aparelho os deslocamentos máximos foram entre 2,53 e 2,89 mm, e as tensões, entre 4.859,7 e 9.157,4 MPa.

**Conclusões:**

Ocorreram maiores concentrações de tensão no mini-implante do que no braço do aparelho expansor. Nas simulações com configuração de três mini-implantes, foi observada sobrecarga de tensão no mini-implante isolado. Os deslocamentos dos mini-implantes e braços do aparelho foram semelhantes em todas as simulações.

## INTRODUCTION

Mini-implant Assisted Rapid Palatal Expander (MARPE) is an appliance for correction of maxillary atresia in adults as an alternative to surgical procedures.^1^ ^-^ ^5^ The mechanical behaviour of the mini-implants and expander appliance during maxillary disjunction is important, especially due to the heavy forces applied to perform the procedure.^6^ The number of mini-implants required for MARPE varies according to the technique and the clinical indication: appliance configurations with two or four mini-implants are more commonly observed.^1^ ^-^ ^4^ ^,^ ^7^ ^,^ ^8^

The finite element method (FEM) is a valuable resource for investigating orthodontic mechanics.^9^ ^-^ ^11^ Stress and strain simulations using the FEM have been shown to be useful for improving the MARPE behavior. For example, hybrid expanders with two mini-implants for anchorage,^9^ the effects on the nasomaxillary complex^11^ and, recently, a comparison between mono- and bicortical anchorage using the MARPE^12^ have been examined.

A clinical reality faced by the orthodontist is the possibility of losing the anchorage mini-implant during the active period of treatment. Faced with situations like that, what should the orthodontist do? The main goal of this study is to determine the stress and displacement of mini-implants and expander appliance by FEM, during rapid maxillary expansion, for different numbers and locations of mini-implants.

## MATERIAL AND METHODS

**Figure 1:**
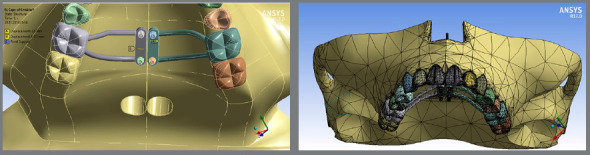
Three-dimensional model and boundary conditions associated with orthodontic appliances.

A maxillary model was produced in SolidWorks^®^ 2015 (Waltham, MA, USA) based on the three-dimensional mesh from a computed tomography image.^13^ After making the maxillary model, the following orthodontic accessories were designed: mini-implants 2 mm in diameter and 10 mm in length (Morelli Ortodontia, Sorocaba, São Paulo, Brazil), and the structure of the appliance to apply expansion displacement. Figure 1 presents the tetrahedral-element three-dimensional mesh model and boundary conditions associated with orthodontic appliances, and the number and position of mini-implants.

The material considered for the mini-implants was Ti-6Al-4V alloy, and stainless steel (AlSl 304) was considered for the expander appliance. The application of conventional mini-implants, and not those indicated to MARPE, may represent a selection bias, however they are appliances previously used to perform the technique.^4^ The elastic modulus of the suture was estimated according to connective tissue and bone values. All the materials simulated in the models were considered to have linear mechanical behavior, using the Young’s modulus (MPa) and Poisson’s coefficient as properties for the simulations. The properties of the materials used are described in Table 1.^9^ ^,^ ^12^ ^,^ ^14^ ^-^ ^16^

**Table 1:** Mechanical properties of the materials used in the models.

Material	Young’s modulus (MPa)	Poisson coefficient
Tooth	20.700	0.30
Periodontal ligament	0.71	0.40
Bone	14.700	0.30
Stainless steel	190.000	0.29
Mini-implant	114.000	0.34

Two types of suture were designed for the simulations: a completely closed suture, representing the phase before suture opening, and another with the hemi-maxillas separated by a 0.5 mm-thick cut.^12^ The open suture did not contain any embolization or interdigitation, so that there is no medial palatal resistance to the expanding force, representing the phase after rupture of the suture.

A 0.5-mm displacement was performed in two 0.25-mm activations in the closed suture model to activate the appliance. In the open suture, a 5-mm displacement of the expander appliance was performed in 20 activations of 0.25 mm.^12^

**Figure 2:**
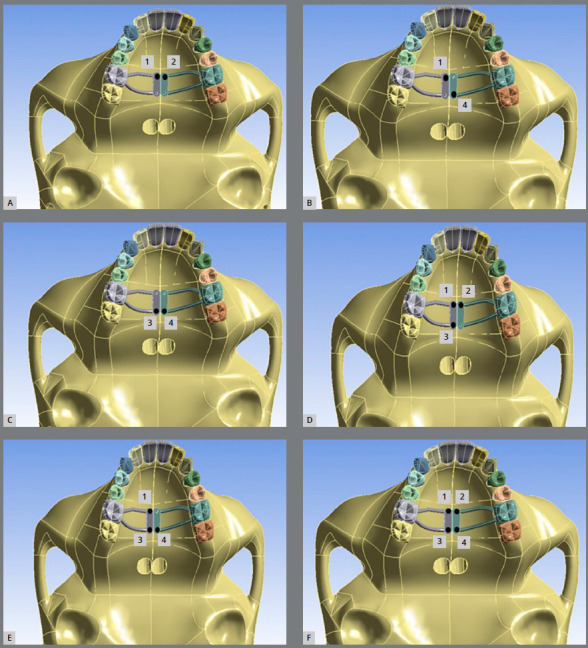
A) Two anterior mini-implants; B) one anterior and one posterior mini-implant on opposite sides; C) two posterior mini-implants; D) three mini-implants, two of which being anterior; E) three mini-implants, two posterior; F) four mini-implants.

The tests were performed on two-, three- and four-anchor mini-implants at different locations, as shown in Figure 2: in A) two anterior mini-implants (1 and 2); B) one anterior and one posterior mini-implant on opposite sides (1 and 4); C) two posterior mini-implants (3 and 4); D) three mini-implants, two of them being anterior (1, 2 and 3); E) three mini-implants, two posterior (1, 3 and 4); F) four mini-implants.

Some boundary conditions were adopted for the simulation. The first was to consider as a fixed support the posterior region of the model, to avoid rotation of the structure when displacements were performed. For the contact between the model elements, the condition of bonded contact was adopted so that there was no type of slip between the parts.

The model was exported to ANSYS R17.0 (Ansys, Inc., Canonsburg, USA), and the geometry was subdivided into 137,817 tetrahedral elements with 251,164 nodes, forming a three-dimensional arranged mesh. The mesh nodes are the connection point between the elements. Each node has a degree of displacement that can be performed in the three dimensions (x, y and z).

## RESULTS

**Figure 3:**
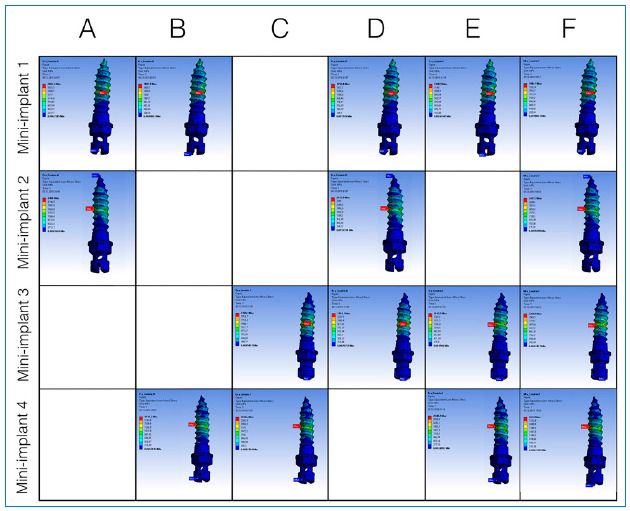
Tension distribution for closed midpalatal suture.

**Figure 4:**
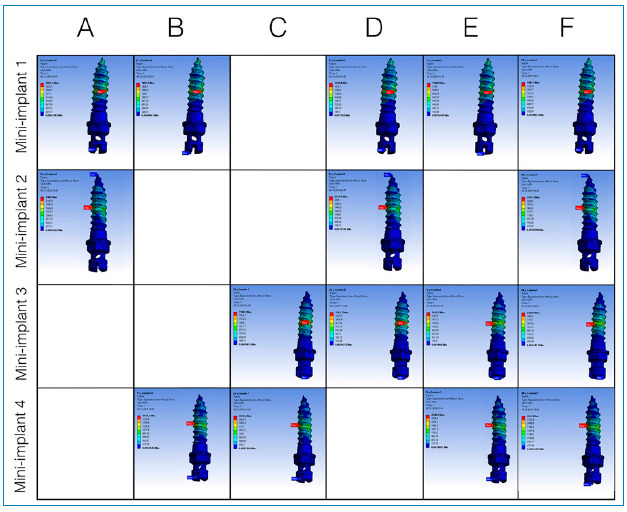
Displacement distribution for closed midpalatal suture.

**Figure 5:**
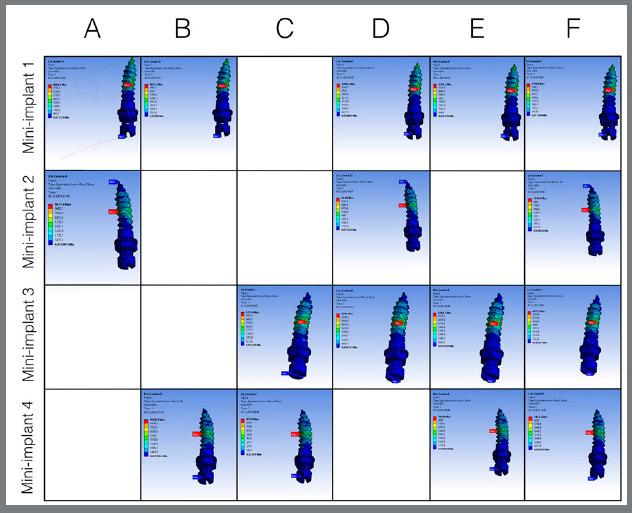
Tension distribution for open midpalatal suture.

**Figure 6:**
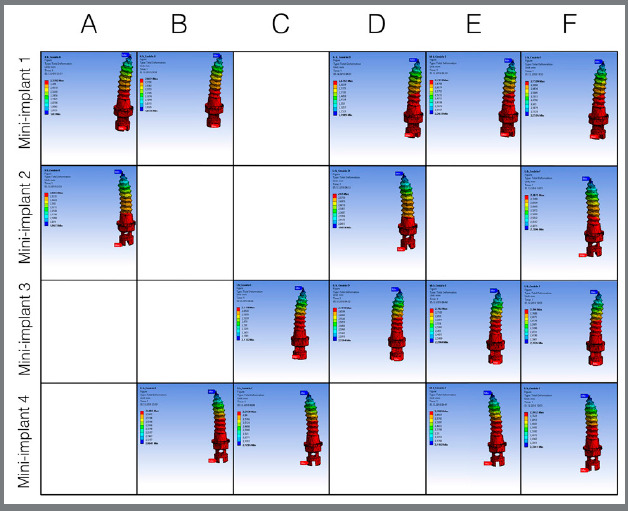
Displacement distribution for open midpalatal suture.

Tables 2 and 3 present the results of the maximum stress (MPa) and displacements (mm), respectively, in the mini-implants and of the expander appliance arm in the closed midpalatal suture model. The maximum stress values were between 1,348.9 and 2,948.2 MPa, as can be seen in Figure 3, which presents the von Mises stress distribution in the mini-implants. The displacements observed were between 0.253 and 0.280 mm, as shown in Figure 4. For the simulations considering the open midpalatal suture model (Tables 4 and 5), the stress values in the mini-implants ranged from 6,190.6 to 10,366 MPa, according to the von Mises stress distribution presented in Figure 5. The displacements observed were between 2.57 and 2.79 mm (Fig 6). Solitary mini-implants on one side showed a significantly higher stress peak than the others, in the simulations of closed and open suture models with three mini-implants (D and E). In the open and closed suture simulations, the group with four mini-implants (F) presented smaller and more balanced values than that with two mini-implants (A, B and C).

**Table 2:** Maximum stress (MPa) in the mini-implants and in the expander appliance arms according to the quantity and location of mini-implants in the closed palatine suture simulation.

	Quantity and location of mini-implants					
	A	B	C	D	E	F
Mini-implant 1	2,062.4	1,803.1		1,656.8	1,348.9	1,982.1
Mini-implant 2	2,440.0			2,917.1		2,475.3
Mini-implant 3			2,199.1	1,391.7	1,943.1	2,208.9
Mini-implant 4		1,939.3	2,659.4		2,948.2	2,624.0
Right arm	814.48	826.81	957.0	1,011.1	1,091.6	892.91
Left arm	1,049.5	1,069.3	1,207.6	664.0	738.52	856.71

**Table 3:** Displacements (mm) of the mini-implants and of the expander appliance arms according to the quantity and location of mini-implants in the closed palatine suture simulation.

	Quantity and location of mini-implants					
	A	B	C	D	E	F
Mini-implant 1	0.275	0.273		0.259	0.259	0.261
Mini-implant 2	0.272			0.280		0.265
Mini-implant 3			0.261	0.257	0.280	0.263
Mini-implant 4		0.261	0.265		0.262	0.266
Right arm	0.272	0.271	0.269	0.281	0.266	0.258
Left arm	0.271	0.271	0.269	0.253	0.256	0.256

**Table 4:** Maximum stress (MPa) in the mini-implants and in the expander appliance arms according to the quantity and location of mini-implants in the simulation of open palatine suture.

	Quantity and location of mini-implants					
	A	B	C	D	E	F
Mini-implant 1	8,664.1	8,623.2		5,788.3	5,765.3	6,190.6
Mini-implant 2	9,677.4			10,366.0		7,449.7
Mini-implant 3			8,351.4	6,269.0	6,268.7	6,631.2
Mini-implante 4		9,428.3	9,513.0		10,016.0	7,453.2
Right arm	4,859.7	7,214.6	7,334.6	5,212.4	7,615.8	5,514.4
Left arm	5352	5,251.6	9,157.4	6,614.0	6,663.6	6,876.3

**Table 5:** Displacements (mm) of the mini-implants and of the expander appliance arms according to the quantity and location of mini-implants in the simulation of open palatine suture.

	Quantity and location of mini-implants					
	A	B	C	D	E	F
Mini-implant 1	2.57	2.60		2.67	2.73	2.75
Mini-implant 2	2.60			2.65		2.78
Mini-implant 3			2.71	2.71	2.76	2.79
Mini-implant 4		2.64	2.70		2.70	2.79
Right arm	2.56	2.63	2.69	2.60	2.70	2.87
Left arm	2.53	2.55	2.71	2.83	2.89	2.86

**Figure 7:**
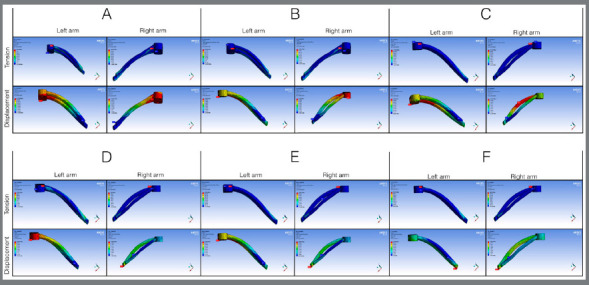
Distribution of tension and displacement in the arms of the expander appliance for closed midpalatal suture.

**Figure 8:**
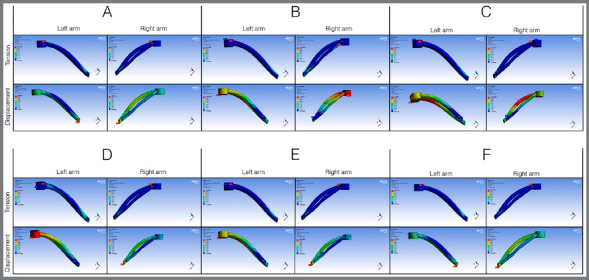
Distribution of tension and displacement in the arms of the expander appliance for open midpalatal suture.

In the arms of the expander appliance, the maximum stress observed was between 738.52 and 1,207.6 MPa, and displacement ranged from 0.256 to 0.281 mm (Fig 7) in the closed palatine suture simulations. For the analyses with open midpalatal suture, the maximum stress observed was between 5,212.4 and 9,157.4 MPa, and maximum displacement was between 2.53 and 2.87 mm (Fig 8). Although the highest concentrations of stress occurred close to the mini-implant contact and expander appliance, the arm segment of the expander appliance near contact with the molar also presented considerable stress in all simulations performed, in both the open suture and closed suture models.

## DISCUSSION

From its inception to the present day, the technique of maxillary expansion supported by mini-implants has undergone modifications either by personal preferences or by the specific needs of the case regarding the number of mini-implants. It is not uncommon to find a palate that does not allow the installation of mini-implants or cases where after installation there is failure in one or more mini-implants. In view of the above, this study aimed to evaluate the stress and displacement of the mini-implants and expander appliance during rapid maxillary expansion, using FEM and varying the quantity and location of the mini-implants.

The mini-implants of the open suture simulation had the highest tensions and displacements when compared to those in the closed suture, despite offering no initial resistance in the medial palatine region. This suggests that the craniofacial bone structure offers important resistance to the displacement applied in the appliance besides, of course, the greater displacement employed. In fact, the variations observed in the forces dissipated by the expander appliance are directly related to the greater or smaller degree of resistance of the craniofacial structures.^6^ Although the mini-implants with the closed palatine suture showed the lowest stress values, the value found is still important, considering the displacement of only 0.5 mm that was performed for the simulation prior to rupture of the suture.

A rapid expansion with two mini-implants for anchorage is preferentially applied in adolescents and in cases of probable lower resistance of the sutures,^7^ ^,^ ^8^ due to the expansion method with four mini-implants supposedly causing opening difficulty to young adults.^1^ ^-^ ^5^ The configuration with four mini-implants in the simulations with open suture showed lower maximum stress values than the configurations with two mini-implants (Table 4, columns A, B, C and F), reinforcing the indication of four mini-implants for MARPE and reserving two mini-implants for cases where the sutures offer less resistance. In the closed suture simulation, similar values were observed in the configurations with two and four mini-implants (Table 2, columns A, B, C, F), probably because the activation displacement was only of 0.5 mm. Additionally, a previous FEM study identified that, in terms of stress distribution and transverse displacement, four mini-implants are preferable.^17^

Deformation of the MARPE anchorage mini-implants was associated with the distance of the force applied to the cortical/mini-implant interface; that is, the further away from the palate the expander appliance, the greater the likelihood of deformation.^1^ Simulations with three mini-implants (Tables 2 and 4, columns D and E) represent the clinical scenario of failure of some mini-implants. Greater stress was observed in the isolated mini-implants, in relation to the symmetrical simulations with two or four mini-implants, indicating that they are appliances with greater overload and the possibility of deformation of the anchorage.

Given the clinical possibility of anchorage failure, the adoption of bicortical anchorage is mandatory for the procedure.^12^ ^,^ ^18^ However, in the case of mini-implant failure, to avoid stress overload in the isolated mini-implant (Table 2 - D and E), it may be necessary to reposition the MARPE in a new location.

Rapid expansion with dental anchoring presents as disadvantages sloping of the anchoring teeth and a reduction in the vestibular alveolar bone crest.^19^ ^,^ ^20^ The incorporation of mini-implants in expander appliances may contribute to forcing delivery to the sutures and a decrease in excessive stress on the buccal plate.^21^ MARPE has the advantage of minimizing these side effects, although the procedure has been questioned in terms of real preservation of the side effects in the molars.^22^ ^,22^ In a clinical study with anchorage of only two mini-implants, a reduction in the buccal cortical surface was observed.^23^ It is possible that the design of the appliance, the quality of the steel and welding, the dimensions of the mini-implants involved, the mono- or bicortical anchorage, the quantity of expansion activations, the age of the patient and the degree of maturation of the midpalatal suture influence reduction of the buccal cortical surface and undesirable vestibular inclination of the molars. In the present study, simulating anchorage with up to four mini-implants, it was observed that the arms of the expander appliance presented stress and displacement in the region near the molar in all groups (Figs 7 and 8), indicating possible side effects, even if of lower intensity.

In this study, the FEM was applied, which generated an approximate computational number of the stress and displacements according to the simulations. It is a method that presents advantages over other research methodologies, to answer the test question, especially for the feasibility of development. However, in the same context it is evaluated as a complementary method of study, and it needs support for its validation of results, with new simulations by FEM or other existing research methodologies. The results obtained here may differ from clinical results because several factors interfere biomechanically in MARPE, such as suture maturity, bone density, biological considerations and anatomy of the palate and adjacent structures.^24^ Therefore, the simulations in this research do not represent all possible clinical situations, and we suggest new computational or mechanical studies to confirm the results.

## CONCLUSION

The results of the FEM simulation indicate that:

1. The arms of the expander appliance presented stress and displacement in the region near the molar.
2. In the scenarios with three mini-implants, the mini-implant isolated on one side received tension overload.
3. The displacements of the mini-implants and arms of the appliance were similar in all tests.
